# An Insiders’ Outside Perspective on the Flemish-Walloon Conflict: The Role of Identification and Disidentification for the German-Speaking Minority

**DOI:** 10.5334/pb.347

**Published:** 2017-11-21

**Authors:** Frank Asbrock, Alain Van Hiel

**Affiliations:** 1 Chemnitz University of Technology, DE; 2 Ghent University, BE

**Keywords:** Identification, disidentification, linguistic conflict, German-speaking community, conflict perception

## Abstract

In this study we analyzed the opinions of citizens of the German-speaking minority in Belgium on the linguistic conflict between the Walloons and the Flemish, as well as their attitudes towards these linguistic communities. We were especially interested in the effects of identification with the local community and disidentification with Belgium. We distributed a survey questionnaire in Eupen, the capital of the German-speaking community, and received replies from 129 inhabitants. Results showed that identification with the German-speaking community was associated with positive attitudes towards the German-speaking community and with demands for more autonomy of the community within the federal Belgian state. Disidentification with Belgium was not positively correlated with these constructive and positive outcomes, but with negative perceptions of all three Belgian communities, the perception of strong conflicts among these communities, and demands for the separation of the Belgian federal state into independent regions. The results are in line with previous research on these processes and point to unique, positive aspects of a strong local identity.

In public perception, especially from an international viewpoint, Belgium is a country which is divided into two linguistic and culturally distinct subgroups, the Flemish and the Walloon communities. This division is prevalent in the present special issue, which includes articles devoted to the linguistic conflict between the French-speaking and the Flemish communities. However, Belgium also has a small German-speaking community with about 76,300 inhabitants, which is located at the Belgian-German border. In the linguistic conflict between the Dutch-speaking and French-speaking communities, the German-speaking community holds an insider’s outside position – although they are part of the superordinate group by being Belgian, their ingroup is not involved in the major societal conflict. Up to this point, the perspective of the German-speaking community has hardly received any scholarly attention, at least among (political) psychologists.

The present study is driven by our interest in the role of identification processes among this rather invisible minority in Belgium. Social identification is an important issue in social psychological research and although it has already been investigated in the context of the linguistic division of the Belgian communities, the existing research has been exclusively focused on the Flemish and Walloon communities as the two major actors in this conflict (e.g., Billiet, Jaspaert, & Swyngedouw, 2012; Klein, Licata, Van der Linden, Mercy, & Luminet, 2012). As a community in Belgium, which has not been directly involved in the conflict, but is nonetheless affected by reforms negotiated by the Flemish and Walloon communities, we consider it important to add a focus on the German-speaking community to the existing body of research. Given this unique insider’s outside perspective, we want to focus our analysis on the relationship between identification processes and attitudes towards the linguistic conflict, as well as towards the other linguistic communities.

The goal of the present paper was twofold. Firstly, we were interested in the relationship between the identification with the German-speaking community and the *dis*identification with Belgium and the perception of the linguistic conflict, the superordinate category (i.e., Belgium), and the other two sub-groups within this category (i.e., the Flemish and Walloons). We build upon a dual identity perspective (e.g., Gaertner, Dovidio, Guerra, Hehman, & Saguy, 2016; González & Brown, 2003), which indicates that minority group members can have a positive minority identity, as well as a positive identity as a member of the superordinate group. Dual identities have been shown to have pacifying effects, as they can reduce ingroup bias (Hornsey & Hogg, 2000), especially for minority groups (González & Brown, 2006). Similar to previous studies of dual identities we were interested in identification processes with subordinate and superordinate groups. In contrast to existing studies, however, we focused on the effects of identification with the minority and *dis*identification with the superordinate group. Recent research suggests that identification and disidentification with a particular group are distinct psychological states, which differentially relate to attitudes and behavior toward this group (Becker & Tausch, 2014). We therefore aimed at analyzing these differential relationships for identification with a subordinate group (the German-speaking community) and disidentification with a superordinate category (the Belgian state). Here, our study adds to the small but growing body of research on disidentification, with a special emphasis on a minority ingroup’s perspective. Secondly, we focused on the interesting but uninvestigated perspective of the German-speaking community on the linguistic conflict and the other communities in Belgium. Before elaborating on our theoretical perspective and reviewing previous research on identification and disidentification, we will briefly introduce the linguistic conflict and the situation of the German-speaking community in Belgium in the following section.

## The historical-political context of the German-speaking community

Belgium’s German-speaking territories have only been part of Belgium since Germany’s defeat in World War I (1914–1918). The treaty of Versailles postulated that the Eupen-Malmédy region and Moresnet had to be handed over to Belgium in order to compensate for the losses and damages caused by the war. Two decades later World War II (1939–1945) began and Germany again occupied these territories. Its inhabitants were considered to be German, and they often referred to themselves as Germans (Wenselaers, 2008). Young men had to join the German armed forces, and not less than 3,200 of them would never return to their homes. Following the defeat of Germany in 1945, the territories again became part of Belgium, and, as a result of alleged collaboration with Nazi Germany, the Belgian and Walloon authorities attempted to de-Germanize the local population (Dewulf, 2009). Almost half of the population faced juridical procedures and one sixth of them were imprisoned (DG, 2016). These procedures were generally seen as exaggerated and unfair and the local population felt that the Belgian authorities showed little understanding for the specific situation of these territories (see van Istendael, 2012; Wenselaers, 2008).

Nowadays, Belgium is a federal state, which is composed of communities and regions. It has three communities: the Dutch, French, and German-speaking Community. The communities have internal autonomy regarding policies related to language and culture in a broad sense. Belgium also consists of three regions: the Flemish, Walloon, and Brussels regions. Regions have a say in economic issues. The German-speaking part of Belgium thus comprises a community in itself, with autonomy in language and cultural issues, but at the same time it is part of the Walloon region, which decides on economic issues (see also, Klein et al., 2012).

The first state reform was implemented in 1973. Over the years, however, not less than six state reforms have been implemented. These reforms have been predominantly driven by the desire for autonomy of the Dutch and French-speaking populations. Yet, it should be acknowledged that the desire for further state reform has more and more become a Flemish demand, while Walloon politicians have been typically opposing such reforms lately due to the apprehension that they might lead to the abolishment of the Belgian state. As a possible side effect, the succession of reforms resulted in increased autonomy for the small German-speaking community (Dewulf, 2009). In the past few years, however, there was a broad consensus among political parties to strive for greater autonomy. This would lead to the transfer of some competences that are currently held by the Walloon Region, such as social policy and (public) transport (Wenselaers, 2008). The German-speaking community’s important political parties are associated with the respective mother parties in the Walloon region, and they constitute local lists of Christian Democrat, Green, Liberal and Socialist parties. The ProDG as the sole regional party emphasizes their independence from the Walloon and Belgian establishment as a selling point to its voters (ProDG, 2016).

## Social Identification and disidentification

Membership in social groups has strong effects on the individual (Tajfel & Turner, 1979). People tend to systematically value others more when they are perceived as being members of the same category. However, not all group memberships are equally important: Individuals can identify more or less strongly with various groups, which in turn affects the social consequences of group membership (Ellemers, Spears, & Doosje., 1999; Leach et al., 2008). One of the best studied consequences of identification with a group is ingroup bias (Tajfel, Billig, Bundy, & Flament, 1971), although other effects have been noted as well. As an example, though minority group members show an increase in perceived discrimination with higher ingroup identification (e.g., Verkuyten & Yildiz, 2007), they also report weaker effects of discrimination experiences on well-being (Schmitt, Branscombe, Postmes, & Garcia, 2014). Moreover, ingroup identification is connected with ingroup support, but not necessarily with outgroup derogation (Brewer, 1999). Research has shown that patriotism, which is a positive attitude toward one’s own country, is either negatively or not at all associated with the derogation of outgroups such as foreigners. Nationalism, which implies dominance and superiority over other nations, is however positively correlated with outgroup derogation (e.g., Wagner, Becker, Christ, Pettigrew, & Schmidt, 2012).

In the case of Belgium, the relevant groups can be nested within a hierarchical structure. Individuals are simultaneously members of a subordinate and superordinate group. One way to effectively deal with this state of group membership is to develop dual identities. The Dual Identity Model is an integration of the Common Ingroup Identity Model (Gaertner et al., 2016), which stresses the importance of recategorization processes that result into a common superordinate group, and the Mutual Intergroup Differentiation Model (Brown & Hewstone, 2005). The Mutual Intergroup Differentiation Model emphasizes the importance of maintaining group boundaries on a subordinate group level. Thus, dual identities allow for the acceptance of the minority group’s distinctiveness while simultaneously being a part of the superordinate majority. These identities reduce intergroup bias in members of minority groups (González & Brown, 2006). Hornsey and Hogg (2000) showed that intergroup bias is lowest when both superordinate and subordinate categories are salient and highest when only the superordinate category is salient.

While dual identities are effective in reducing intergroup bias, minority group members do not always identify with their ingroup as well as with the superordinate group. In a study including samples of Turkish-Dutch Muslims, Verkuyten and Yildiz (2007) showed that ethnic and religious identification was negatively and positively correlated with identification and disidentification with the superordinate group (i.e., the Dutch), respectively. Disidentification describes the psychological phenomenon of belonging to a group one does not want to belong to and which poses a threat to one’s identity (Becker & Tausch, 2014; Dean, 2008). Various studies did not distinguish between non-identification or low identification and disidentification (e.g., Becker, Tausch, Spears, & Christ, 2011; Jasinskaja-Lathi, Liebkind, & Solheim, 2009). Nevertheless, a growing body of research indicates that non- and disidentification are distinct psychological states. In a recent review, Becker and Tausch (2014) described disidentification as a multi-dimensional construct, comprising detachment from the group, dissatisfaction with membership, and perceived dissimilarity to other group members. Various researchers have demonstrated that disidentification indeed differs from non-identification, and therefore disidentification is not the mere opposite of identification with a group (e.g., Becker & Tausch, 2014; Ikegami, 2010; Long & Spears, 1997; Verkuyten & Yildiz, 2007). Unlike non-identification, which is a rather neutral process without emotional involvement towards the respective group, disidentification implies a high affective investment and an active separation from the group (Becker & Tausch, 2014; Dean, 2008). Ikegami and Ishida (2007) showed that disidentification has stronger relationships with negative evaluations of the ingroup compared to low identification. Also, Becker and Tausch (2014) demonstrated that disidentification correlated stronger with negative behavioral intentions, as well as negative emotions, towards the ingroup than identification. Identification, however, showed stronger correlations with positive emotions and behavioral intentions.

In the present study, we aimed at examining to what extent members of the German-speaking community identify with their ingroup and simultaneously disidentify with the superordinate group in the form of the federal Belgian state. Our research focus differs from previous studies, which analyzed dual identities (but not disidentification; e.g., González & Brown, 2006; Hornsey & Hogg, 2000) or ethnic/religious identification and national disidentification (Verkuyten & Yildiz, 2007). We argue that ingroup identification does not necessarily lead to disidentification with the superordinate group (see Verkuyten & Yildiz, 2007). Instead, as suggested by Becker and Tausch (2014), we expected identification and disidentification to be uncorrelated. For our present research, this means that a high identification with the German-speaking community does not indicate disidentification with Belgium. Both dimensions should rather be independent and therefore uniquely predict perceptions of and attitudes toward the linguistic conflict in Belgium as well as the groups involved.

## Hypotheses

Regarding the identification with the German-speaking minority, we hypothesized that a higher identification is related to more positive attitudes towards the ingroup (Hypothesis 1). In line with previous research (Becker & Tausch, 2014), we hypothesized that disidentification with Belgium is associated with a negative perception of the two main groups involved in the linguistic conflict (Hypothesis 2a). As the Walloon and Flemish communities are both part of the superordinate group, a general negative evaluation of Belgium should also result in negative evaluations of its subgroups. Moreover, we expected disidentification to be positively associated with the perception of a stronger linguistic conflict between the communities (Hypothesis 2b). As identification focuses on the ingroup and does not necessarily imply outgroup derogation (Brewer, 1999), we expected the effects of disidentification to be stronger than any effects of identification with the German-speaking community on attitudes towards the two main groups involved in the conflict (Hypothesis 3a) as well as on the perception of the conflict itself (Hypothesis 3b).

The next hypothesis builds upon the outsider perspective of the German-speaking community. As outlined above, the German-speaking community is not directly involved in the linguistic conflict. Higher identification with the subordinate ingroup, as well as stronger disidentification with the superordinate Belgian state, should intensify this outsider perspective. We therefore predicted that both disidentification and identification are more strongly correlated with the perception of the linguistic conflict as a conflict between the Walloons and the Flemish community than as a conflict between the German-speaking community and the two other communities (Hypothesis 4).

In sum, we predicted that the concepts of identification with the minority ingroup and disidentification with the superordinate group are psychologically distinct and should therefore show differentiated relations to conflict perceptions and with group evaluations. Identification and disidentification can play distinct roles in the perception of the linguistic conflict in Belgium and allow for a differentiated analysis of the perspective of members of the German-speaking community in Belgium on the linguistic conflict.

To our knowledge, there is hardly any psychological research on the perspective of the German-speaking community on the linguistic conflict and the groups involved. As outlined above, the German-speaking minority profited from the reforms driven by the Dutch and French-speaking communities (Dewulf, 2009). Recently, political parties showed a broad consensus regarding the strive for even greater autonomy. Therefore, we were interested in the relationships of identification with the German-speaking minority and disidentification with Belgium with attitudes towards political reforms regarding the future of the three communities and the Belgian federal state. A related aspect of interest was the relationships of identification and disidentification with political party preferences. In contrast to the majority of the important parties in the German-speaking community, which are related to their mother parties in the Walloon region, the ProDG is a strong regional party, which most clearly strives for more autonomy of the German-speaking community. Therefore, a positive correlation between identification with the German-speaking community and preference for ProDG can certainly be expected. Although we did not derive specific hypotheses about these attitudes and party preferences, we were interested in the associations from an exploratory perspective.

We aimed at testing our hypotheses regarding identification and disidentification in a survey administered to households in the German-speaking community.

## Methods

### Participants and procedure

Data were collected in Eupen, the capital of the German-speaking region in Belgium. We distributed 1,000 envelopes containing the questionnaire in the inhabitants’ mailboxes in various streets in and nearby the city center. An accompanying letter introduced the study stating that we were interested in the beliefs and attitudes about a number of political issues of people living in the German-speaking community. The addressees were invited to complete the questionnaires, which they could return without any costs. In order to increase the response rate participants could win a gift certificate of 100 euro, per 50 returned envelopes. A total of 129 questionnaires were returned. Seven persons with German nationality as well as one person with British nationality were excluded from the analysis, leaving a total sample of 121 participants (113 with Belgium nationality, 8 unknown), including 76 men and 45 women, with a mean age of 49 years (*SD* = 17.4). A total of 117 participants stated that German was the language they used at home. A majority of the participants (*N* = 73) completed higher education and 43 participants completed secondary education. One person only finished primary education. Most of the respondents worked fulltime (*N* = 74) and a significant number of participants were retired (*N* = 24). Also included were a number of part time workers (*N* = 7), unemployed people (*N* = 4), students (*N* = 5), and one housewife.

### Measures

**Identification with the German-speaking community.** We assessed identification with 14 items of Leach et al.’s (2008) instrument, which measures identification in terms of group solidarity, satisfaction, centrality, self-stereotyping, and the perception of in-group homogeneity by means of five-point Likert scales (1 = totally disagree; 5 = totally agree). The German version of the scale (see, Becker & Tausch, 2014) showed sufficient internal consistency (*M* = 3.52; *SD* = .88; α = .94). Sample items include “I feel committed with the German-speaking community” and “I often think about the fact that I am a member of the German-speaking community.”

**Disidentification with Belgium.** Disidentification was measured with ten items from a scale developed by Becker and Tausch (2014) using a five-point Likert scale (1 = totally disagree; 5 = totally agree). The scale assesses group detachment, dissimilarity, and dissatisfaction, and showed good internal consistent scores (*M* = 1.34; *SD* = .46; α = .79). Sample items are “I feel a distance between myself and the Belgians” and “I have nothing in common with most Belgians.”

**Party preference.** Participants indicated how much they agreed with the political program of the CSP (Christian Democrats; *M* = 3.89; *SD* = 1.76), Ecolo (Green party; *M* = 3.77; *SD* = 1.60), SP (Social Democrats; *M* = 3.21; *SD* = 1.62), PFF (Liberal party; *M* = 3.41; *SD* = 1.73), ProDG (Regional party *M* = 3.78; *SD* = 1.84), or other parties (*M* = 2.54; *SD* = 1.95) via Likert scales from 1 (not at all) to 7 (fully).

**Attitudes toward members of different language groups and Germans.** Participants provided thermometer ratings for various groups (0 = cold, negative; 100 = warm, positive). Mean ratings were 65.25 (*SD* = 22.38) for the Wallons, 66.64 (*SD* = 21.52) for the Flemish, 75.25 (*SD* = 18.15) for the German-speaking community, 60.61 (*SD* = 22.68) for inhabitants of Brussels, and finally 60.76 (*SD* = 22.44) for Germans living in Germany.

**Linguistic conflict perceptions.** Participants rated the amount of conflict between the different language groups on a thermometer ranging from 0 (peaceful, no conflict) and 100 (strong conflict). The means values were 66.27 (*SD* = 23.20) for the conflict between Flanders and Wallonia, 43.28 (*SD* = 25.38) for the conflict between Wallonia and the German-speaking community, and 30.60 (*SD* = 22.94) for the conflict between Flanders and the German-speaking community.

**Political reform.** We asked participants to react to seven statements regarding possible state reforms of Belgium. They indicated the extent to which they desired the implementation of these reforms on a scale from 1 to 5 (1 = not desirable at all; 5 very desirable). The items were: (1) The organization of the federal Belgian state will remain unchanged (*M* = 3.27; *SD* = 1.37); (2) The language groups will acquire more autonomy, but the Belgian state will remain (*M* = 3.73; *SD* = 1.24); (3) Belgium will be divided into independent regions (*M* = 1.30; *SD* = .88); (4) The German-speaking community will become a distinct region (*M* = 3.00; *SD* = 1.49); (5) The German-speaking community will join Germany (*M* = 1.27; *SD* = .80); (6) The German-speaking community will join the Walloon region (*M* = 1.41; *SD* = .89); and (7) The German-speaking community will join the Flemish region (*M* = 1.49; *SD* = 1.02).

## Results

We tested our hypotheses in separate path models for conflict perception and attitudes toward the different language groups. In addition, we ran separate path analyses for party preferences and attitudes toward political reform. We controlled for age, gender, and education for all analyses and used robust maximum likelihood estimators. As implemented in Mplus (Muthén & Muthén, 1998–2015) we used full information maximum likelihood to handle missing data (Schäfer & Graham, 2002). Table 1 shows intercorrelations of all variables for hypothesis testing and demonstrates that disidentification with Belgium is not significantly correlated with identification with the German-speaking community (*r* = .08, *p* = .38).

**Table 1:** Correlations.

	Ident	Con W-F	Con W-G	Con F-G	FT Wall	FT Flem	FT GSC	FT Ger	FT Bru	age	gender	edu

Disident	.08	.20*	.32***	.24*	–.48***	–.37***	–.21*	.07	–.42***	–.03	.00	–.05
Ident		.25**	.14	.00	–.04	.09	.35***	.23*	.01	.04	.01	–.10
Con W-F			.53***	.31**	–.04	–.09	.20*	.10	.04	–.34***	.19*	–.04
Con W-G				.50***	–.26**	–.03	.05	.13	–.14	–.05	.07	–.11
Con F-G					–.21*	–.38***	–.28**	.04	–.29**	–.05	.09	–.22*
FT Wall						.48***	.41***	.20*	.68***	–.13	.04	.08
FT Flem							.57***	.31**	.47***	.11	–.03	.01
FT GSC								.42***	.58***	–.23*	.15	.22*
FT Ger									.31**	.04	.07	–.10
FT Bru										–.18*	.06	.27**
age											–.22*	–.32***
gender												–.08

*N* = 121. Disident = Disidentification, Ident = identification, Con W-F = Conflict between Walloons and Flemish community, Con W-G = Conflict between Walloons and German-speaking community, Con F-G = Conflict between Flemish community and German-speaking community, FT Wall = Feeling thermometer Walloons, FT Flem = Feeling thermometer Flemish community, FT GSC = Feeling thermometer German-speaking community, FT Ger = Feeling thermometer Germany, FT Bru = Feeling thermometer people from Brussel, gender (1 = female, 2 = male). ***p < .001, **p < .01, *p < .05.

**Figure 1:**
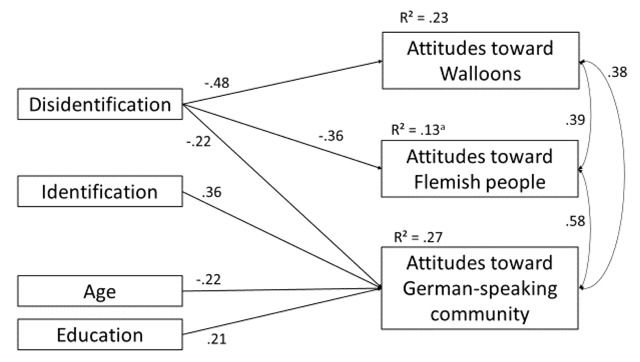
Path model predicting attitudes toward outgroups and ingroup. All coefficients are standardized estimators. All reported effects are significant (*p* < .05). *^a^p* = .14.

**Attitudes.** We regressed attitudes toward the Walloons, the Flemish community, and the German-speaking community simultaneously on disidentification with Belgium, identification with the German-speaking community, and the control variables. Figure 1 shows the model including the significant paths only. The model shows a good fit (χ^2^ = 6.612, *df* = 6, *p* = .36; CFI = .995, RMSEA = .030, SRMR = .034). In line with Hypothesis 1, identification with the German-speaking community was positively associated with attitudes towards the ingroup (β = .36, *p* < .001), but not with attitudes toward the other two groups. As predicted in Hypothesis 2a, disidentification had a negative effect on attitudes towards the Walloon (β = –.48, *p* < .001) and the Flemish communities (β = –.36, *p* = .003), but also on attitudes toward the German-speaking community (β = –.22, *p* = .01). These results support our assumption that disidentification with Belgium has a stronger negative effect on attitudes towards the Walloon and Flemish communities than identification with the German-speaking community (Hypothesis 3a).^1^

**Figure 2:**
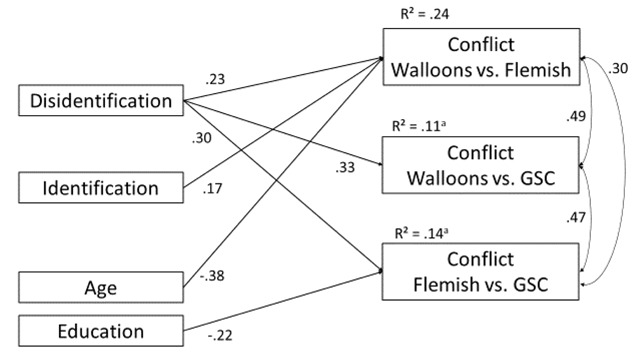
Path model predicting perceptions of linguistic conflict. GSC = German-speaking community. All coefficients are standardized estimators. All reported effects are significant (*p* < .05). *^a^p* < .09.

**Conflict perceptions.** We tested our predictions regarding conflict perception in a path model using the same predictors as for attitudes and included the perception of the linguistic conflict between the Walloon and Flemish communities, Walloons and the German-speaking community, and the Flemish and German-speaking communities simultaneously as dependent variables. Figure 2 shows the model with significant paths only, which indicates a good model fit (χ^2^ = 4.470, *df* = 6, *p* = .61; CFI = 1.000, RMSEA = .000, SRMR = .037). As predicted in Hypothesis 2b, disidentification with Belgium was associated with stronger conflict perceptions between the Walloons and the Flemish community (β = .23, *p* = .001), the Walloons and the German-speaking community (β = .33, *p* < .001), and the Flemish and the German-speaking communities (β = .30, *p* = .02). Identification had an effect on perception of the conflict between the Walloon and the Flemish community (β = .17, *p* = .02). Supporting Hypothesis 3b, the relations between disidentification and conflict perception between the German-speaking community and the two other regions were stronger than those between identification and these conflict perceptions. To test the predicted difference between the relationships of disidentification and identification with conflict perception between the Walloon and the Flemish community, we compared a model where the paths in question were constrained to be equal with our unconstrained model. We used a χ^2^-difference test to evaluate whether the models differed significantly (Satorra & Bentler, 2001). The test revealed a marginally significant difference between the paths of disidentification and identification on conflict perception between Walloons and the Flemish community (Δχ^2^_SB_ = 3.734, *df* = 1, *p* = .053), indicating that disidentification had a marginally stronger relationship with conflict perception between the Walloon and the Flemish community than identification.

Hypothesis 4 was only partially supported. As expected, identification showed a stronger relationship with conflict perceptions between the Walloon and Flemish communities than with the two other conflict perceptions, however the effect of disidentification on the perception of the Walloon-Flemish conflict did not differ from the two other conflicts. A χ^2^ difference test between the unconstrained model and a model with all three paths from disidentification constrained to be equal did not reveal a significant difference (Δχ^2^_SB_ = 1.568, *df* = 2, *p* = .46).^2^

**Party preferences.** We analyzed the relationship of identification and disidentification with preferences for political parties by simultaneously regressing the preferences for CSP, Ecolo, SP, PFF, and ProDG on disidentification, identification, and the control variables. The model with significant paths only showed a good model fit (χ^2^ = 5.184, *df* = 4, *p* = .27; CFI = .984, RMSEA = .052, SRMR = .039). Identification with the German-speaking community was associated with preference for CSP (β = .22, *p* = .01), Ecolo (β = .27, *p* = .003), SP (β = .22, *p* = .02) and, most strongly, for ProDG (β = .56, *p* < .001). Disidentification with Belgium showed negative effects on preference for CSP (β = –.15, *p* = .02), Ecolo (β = –.17, *p* = .04), and SP (β = –.23, *p* = .003). As the only control variable, age was associated with preference for CSP (β = .33, *p* < .001). No other effects reached significance.

**Political reform.** As stated in the description of the measures, the mean values for unifications of the German-speaking community with Germany, the Walloon or Flemish communities, as well as the preference for independent regions, were very low and indicated no strong wish for these options in general. A path analysis with simultaneous regression of all political reform items on the predictor variables showed very different results for disidentification with Belgium and identification with the German-speaking community. The model with significant paths only showed a good model fit (χ^2^ = 8.402, *df* = 10, *p* = .59; CFI = 1.000, RMSEA = .000, SRMR = .041). Disidentification, but not identification, was associated with opposition to an unchanged organization of the federal Belgian state (β = –.22, *p* = .003), with preference for a division of Belgium into independent regions (β = .34, *p* < .001), and with preference for a unification of the German-speaking community with Germany (β = .48, *p* < .001). Identification, but not disidentification, was associated with a preference for more autonomy of the German-speaking community (β = .34, *p* < .001), and with opposition to a unification of the German-speaking community with the Walloon region (β = –.20, *p* = .03). Both disidentification (β = .17, *p* = .03) and identification (β = .34, *p* < .001) were associated with preference for the German-speaking community as a distinct region in Belgium. Age was associated with preference for an unchanged organization of the federal Belgian state (β = .31, *p* < .001).

## Discussion

In the present paper we aimed at analyzing the perspective of an often neglected third party in the Belgian linguistic conflict – the perspective of the Germany-speaking minority. Although this small community living in the East of Belgium is not directly involved in the conflict, it is nevertheless affected by political decisions by the two strong communities and the federal Belgian state. Therefore, we considered the view of members of this community on the Belgian linguistic conflict to represent an insiders’ outside perspective. This very specific setting allowed us not only to conduct one of the first studies in political psychology on the specific attitudes and perceptions of the German-speaking minority, but also provided us with the opportunity to analyze more general processes of identification and disidentification with a minority ingroup and a superordinate group. Our research supports the distinction between disidentification and identification as separate constructs (Becker & Tausch, 2014; Verkuyten & Yildiz, 2007). Extending previous research (Becker & Tausch, 2014), we demonstrated that identification with the German-speaking community is correlated with a multitude of positive attitudes toward this ingroup, but not with such attitudes toward outgroups within the same superordinate category. Our results thus indicate that identification constitutes a positive variable. Conversely, disidentification with the superordinate group was related to *negative* attitudes toward the respective outgroups within the entire hierarchy, therefore substantiating the theoretical argument that disidentification is not the mere opposite of identification and therefore differs from non-identification. Our results indicate that disidentification is rather a negative motivational state, expressing the active separation from an ingroup. This destructive component of disidentification is illustrated by the positive relationship between disidentification and support for abolishing the Belgium federal state.

Additionally, disidentification and identification were related to the perception of the linguistic conflict in different ways: While disidentification was related to the perception of stronger conflicts among the three communities, identification was positively correlated with the perception of a strong conflict between Walloons and the Flemish community only. Again, this result points to different motivational states expressed by identification and disidentification processes and to the necessity of separating these concepts.

An important aspect in our study was the simultaneous analysis of identification with a minority ingroup and disidentification with a superordinate group. Previous research on dual identities indicates that identification with both the ingroup and the superordinate group can reduce intergroup bias (González & Brown, 2006; Hornsey & Hogg, 2000), but only little is known about identification and disidentification with different groups within the same hierarchy. As expected, identification with the German-speaking community was not correlated with disidentification with Belgium. However, a negative relationship may be likely when the superordinate group poses a threat to the ingroup (like in the case of Turkish-Dutch Muslims; Verkuyten & Yildiz 2007), while a positive relationship, namely a dual identity, is a likely outcome of a successful re-categorization process (González & Brown, 2006). However, as attested by the present results, it is also possible that both identity concepts are almost unrelated.

Indeed, the present pattern of results points to an interesting case of dual identity. Based on the Politicized Collective Identity Model (Simon & Klandermans, 2001), Simon and Grabow (2010) showed that dual identities as members of a minority and a superordinate majority are positively correlated with supporting political demands and actions within the limits of normative acceptance. For the present context, this means that the identification with the German-speaking community might go along with a critical, but constructive view on the federal state as well as its constituting regions. Our findings support this view by showing that identification with the German-speaking community is positively related to demands for more autonomy for the region within a federal, Belgian state, but not with derogation of the other regions or the perception of strong conflicts among all regions. This negative perception is only related to disidentification with Belgium.

Positive associations between identification with the German-speaking community and party preferences provide further support for this idea, especially with a preference for the ProDG, which aligns well with the strong relation of identification and preference for autonomy of the German-speaking community. Identification was also related to increased preference of national parties such as Ecolo and the Socialist Party, although these correlations were of lesser magnitude than for ProDG. However, this indicates a preference for political action within the limits of normative acceptance. Conversely, disidentification was negatively related to preference for the national parties CSP, Ecolo, and SP, and unrelated to preference for ProDG, which further underlines the differentiation of identification and disidentification processes. This latter finding supports the notion that disidentification with the Belgian state does not lead to support for a party that demands more autonomy of the German-speaking community within the federal state.

In a broader theoretical sense, our findings add to the growing body of research on the important differentiation of identification and disidentification for fully understanding positive and negative aspects of ingroup perception.

In the remainder of the discussion we first focus on the extreme low levels of disidentification with Belgium, which we consider noteworthy, given the German-speaking territories’ short common history as a Belgian territory. Second, we discuss the policy implications of increased identification with the German-speaking community, putting the present results in the context of possible effects of identification in the other language communities. Finally, we pay attention to the beneficial, constructive role identification may play in Belgian policies.

### The last Belgians

Considering the present circumstances of a fierce debate between the dominant communities constituting the large majority of the Belgian population and political parties (especially on the Flemish side) which have either asked for or directly suggested the abolishment of Belgium, it is noteworthy that a small minority group expresses such a low level of disidentification with the Belgian state, close to the scale’s minimum value of 1 (*M* = 1.34, *SD* = .46) and significantly different from the scale midpoint of 3, *t*(118) = –.38.91, *p* < .001. In popular literature, the patriotism of the German-speaking citizens has been coined as ‘the last Belgians’ (van Istendael, 1989; Wenselaers, 2008). Another reason designating this finding as remarkable is the fact that these territories have been subject to rather fierce ‘de-Germanization’ policies, especially after the Second World War and the alleged collaboration of German-speaking citizens (although they were often forced to join the German troops; see Dewulf, 2009). Finally, a third reason for the noteworthiness of this the observation is that in less than a century, these territories have shifted from Germany to Belgium two times from one direction to the other and vice versa. Inhabitants of neutral Monseret – a small portion of these territories – have even changed nationality five times and some citizens have served in two different armies (see, Van Reybroeck, 2016).

Another important related observation is that, although the identification level with the German-speaking community is fairly high (*M* = 3.52, *SD* = .88), at the same time this score is not too far from the scale’s theoretical midpoint of 3, even though the difference is significant, *t*(119) = .6.47, *p* < .001. A possible reason for this is that the German-speaking territory itself is rather heterogeneous. The northern territories around Eupen have been part of the duchy of Limburg, while the southern territories around Sankt-Vith have been part of the duchy of Luxemburg. Moreover, the local languages are different and there has not been much contact between the northern and southern parts because of the presence of a desolate area in between them (Fonteyn, 2013). In an interview, Karl-Heinz Lamberts – the former minister-president – declared that the German-speaking community needs to have an identity, or put otherwise, is in search of an identity as a community (in, Wenselares, 2008). This lack of a strong basis for a common German-speaking identity may be beneficial to people’s identification with the superordinate structure.

### Identification and attitudes about separation and the other language communities

We consider the present findings to be politically relevant: The increased desire for more autonomy among citizens of the German-speaking community does not imply an increased desirability to abolish Belgium as the superordinate state structure (*r* = –.01, *p* = .89). Support for these two policies is fueled differently by identification and disidentification processes: While identification with the German-speaking community was related to support for more autonomy, but not to abolishing the superordinate Belgium state, disidentification with Belgium was related to support for abolishing the superordinate state, but not for more autonomy. Along similar lines, ProDG aims at strengthening the German-speaking region, while the abolishment of the Belgian state is not included in their party platform (ProDG, 2016). It remains open whether similar results would be obtained in the other Belgian language communities with regard to their attitudes about the Belgian state and the other language communities.

With respect to their attitudes about the Belgian state, one would be inclined to expect another pattern of results, especially for citizen living in the Flemish community. Indeed, Flemish regionalists tend to have negative attitudes towards the Belgian state and they even have repeatedly stated that the disintegration of Belgium is their final goal (see, Rihoux, Dumont, De Winter, Deruette, & Bol, 2009). All of this is of great concern for most French-speaking politicians and citizens, as a Flemish regionalist party currently is the largest Belgian party in terms of electoral support, and it is even a leading party in the current national government. Due to the political platform of these regionalist parties and their large share of support, identification among Flemish citizens at the local level might correlate positively with disidentification at the Belgian level, and both these identification processes might relate to the wish to abolish the Belgian state. Conversely, in the Walloon community the movement (i.e., ‘Rassemblement Wallonie-France’) for the (re-) unification of Wallonia with France attracts only 1% of the votes, while no other regional parties advocate the abolishment of the Belgian state. In the Walloon community, identification with the local level should thus not be expected to go along with disidentification with the Belgian level. In this respect, previous studies have revealed that identification with the Walloon and Belgian levels correlate positively (Billiet et al., 2003; Billiet et al., 2012).

With respect to their attitudes regarding the other language communities, regionalists of both the Dutch- and French-speaking communities tend to have a negative attitude towards (citizens of) the other region (see, Nuttin, 1976) as they perceive themselves to be victims of the respective other community (Klein et al., 2012). Hence, unlike the German-speaking citizens, identification processes in Dutch- and French-speaking citizens might go together with negative perceptions of the other communities.

### Limitations and directions for future research

To our knowledge, the present study is the first to analyze the German-speaking community’s perspective on the linguistic conflict in Belgium under consideration of identification and disidentification processes. We were able to show unique relations of identification with the ingroup and disidentification with Belgium, the superordinate group, with perceptions of the Walloon and Flemish communities, of the linguistic conflict, as well as with political attitudes and demands. Still, we are aware of some limitations of our study, which should be addressed in future research. First, we did not include measures of identification and disidentification for both the minority and superordinate groups. The selection of only two measures instead of the full quadrant was informed by the research question at hand, that is, the explicit aim to explain linguistic division rather than to search for elements that bring unification and harmony among the language groups. However, as we outlined above, it seems likely that members of the German-speaking community experience dual identities, which, in turn, should predict a critical, but constructive relation to the federal Belgian state as well as the local community. Even though our findings support this view, we were not able to directly test it. We consider this a very important and fruitful research avenue, as it is not only of theoretical interest but might indicate a special status of the German-speaking community in perceptions of Belgium and commitment to the federal state. This possible dual identity might differentiate the German-speaking community from the other communities, for which a high identification with the local community might go along with low commitment to the federal state and/or a negative attitude towards the other language group.

Second, our study design is correlational and does not allow for any conclusions about causality. Even though experimental evidence suggests that identification and disidentification predict attitudes and bias (e.g., Becker & Tausch, 2014), we cannot confirm this causal relationship with our data. Consequently, we need experimental and longitudinal studies to replicate and extend our findings.

Third, our sample is not representative for the German-speaking community in Belgium. All participants were residents of Eupen. However, our sample shows a quite large distribution of age, education and occupation.

## Conclusion

The perspective of the German-speaking minority in Belgium has not yet been in the focus of any political psychological research. Even though the German-speaking minority has an outsider position in the linguistic conflict, identification with the community and disidentification with the federal state have strong implications for the perception of this conflict. We have shown that identification with the ingroup has rather constructive and positive effects, while disidentification with the Belgium state is correlated with negative attitudes toward the other Belgian communities as well as with support for policies that aim to abolish the Belgian state. Identification and disidentification thus have differential implications, attesting to the necessity to differentiate between these two processes.

## Footnotes

For exploratory reasons, we also tested if the interaction of disidentification with Belgium and identification with the German-speaking community predicts attitudes toward the different groups. Explained variance did not increase due to including the interaction term (*p*s > .44).

Again, including the interaction of disidentification with Belgium and identification with the German-speaking community did not explain any additional variance (*p*s > .24).
